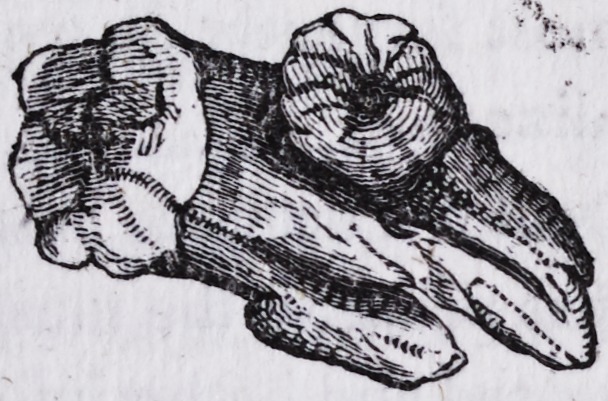# Case of Exostosis, and Osseous Union of Two Molar Teeth

**Published:** 1840

**Authors:** G. Merryman

**Affiliations:** Baltimore


*For the American Journal of Dental Science.*

Messrs. Editors :—

The osseous union of the teeth has been of too frequent occurrence, and its existence too well established to need any confirming evidence to settle the question, which, until lately, has been a mooted one.

But as there is a peculiarity in the specimen which I have in my possession, differing in its appearance, from any that I have seen recorded, I have thought it would be interesting to the profession, to see a drawing with a history of the case.

About two years since, a gentleman (aged about thirty-five) called upon me for the purpose of having his teeth examined; at the same time complaining of a dull, heavy pain in the upper part of the face, extending back, in the direction of the temple.

In examining the teeth, I found the second superior molar considerably protruded from the alveolar cavity, and an accumulation of hard black tartar upon its fangs, with a considerable quantity of fetid pus discharged from the cavity. The dens sapientiæ was apparently sound and healthy,

---

Fig. 2. This is the plate referred to, made of platina, bent on itself and mounted with two golden caps, intended to strike on the interior and laterally of the incisor and canine tooth of the upper maxillary. By the use of these plates, a forward and lateral motion was given to the jaw, and the deformity thus remedied by its use.

2

and had been in position some five or six years. Supposing it to be a case of necrosis, I advised the removal of the tooth. In performing the operation, I found the posterior one to be sensibly affected and remarked that they were connected; but deeming it of more importance to remove both, than suffer the one to remain, the operation was performed, and the teeth exhibited the following curious phenomenon.

The root of the dens sapientiæ, was of the conical form, adhering to the external posterior fang of the second molar; and also, upon the posterior portion of the internal fang, the appearance of each exhibiting, by its singular and unnatural enlargement, a clear and decided case of exostosis.

The abscess containing the matter, was situated between the external posterior and internal fangs, lying upon the superior surface of the crown and by morbid action, producing a caries extending to the dental cavity.

The striking difference between this case and those recorded by Mr. Fox, Bell, Parmly, and others, is this: — In those reported by them, the union has been produced during the ossification of the teeth. And in the one under consideration, it has been effected by a deposition of ossific matter after the formation and growth of the teeth upon their fangs brought in contact by the absorption of the transverse portion of the alveolar process.

Respectfully,

G. MERRYMAN.

*Baltimore, June 6th,* 1840.

## Figures and Tables

**Figure f1:**